# The Emerging Role of Icosapent Ethyl in Patients with Cardiovascular Disease: Mechanistic Insights and Future Applications

**DOI:** 10.3390/jcm12113758

**Published:** 2023-05-30

**Authors:** Ashish Gupta, Mohammad Alkhalil

**Affiliations:** 1 Cardiothoracic Centre, Freeman Hospital, Newcastle upon Tyne NE7 7DN, UK; ashish.gupta@nhs.net; 2 Translational and Clinical Research Institute, Newcastle University, Newcastle upon Tyne NE1 7RU, UK; 3 Department of Cardiothoracic Services, Freeman Hospital, Freeman Road, Newcastle upon Tyne NE7 7DN, UK

**Keywords:** eicosapentaenoic acid, docosahexaenoic acid, triglycerides, polyunsaturated fatty acids, VLDL, coronary artery disease, atherosclerosis

## Abstract

Omega-3 polyunsaturated fatty acids (PUFAs) were early established as therapeutic option for patients with high triglyceride levels. Their effects on lipoprotein particles, including a reduction in very low-density lipoprotein and a shift from small to large low-density lipoprotein, is increasingly recognised. This is coupled with their ability to be incorporated within the cellular membrane, leading to plaque stability and anti-inflammatory effects. Nonetheless, recent clinical trials have not been consistent in demonstrating the potential cardioprotective effects of omega-3 fatty acids. This is despite the circumstantial evidence from imaging studies illustrating the stabilising effects on atherosclerotic plaques and slowing of plaque progression. In this article, we will review the effects of omega-3 fatty acids, eicosapentaenoic acid (EPA) and docosahexaenoic acid (DHA), on lipid biomarkers, atherosclerotic plaque features, and clinical outcome studies and provide a mechanistic role in managing residual risk of atherosclerosis. This will provide better insight into the inconsistency of the recently reported clinical outcome studies.

## 1. Introduction

Patients with established atherosclerotic cardiovascular disease (ASCVD) remain at increased risk of future adverse cardiovascular events despite being on guideline-directed optimal medical treatment [1]. Contemporary data revealed that more than 20% of patients with ASCVD re-present with a second cardiovascular event over 5 years follow-up [2,3]. This risk is evident despite a large proportion of patients receiving anti-platelets, anti-hypertensive and low-density lipoprotein cholesterol (LDL-c)-lowering treatments. This residual risk is also heterogenous, and targeting modifiable atherosclerotic disease characteristics such as inflammation or thrombosis would result in improved patients’ clinical outcomes [4,5,6,7,8]. However, an unselected approach was associated with significant side effects challenging the routine use of potent anti-inflammatory or anti-thrombotic treatments [4,5,6]. Importantly, certain groups of patients sustained larger clinical benefits and provide a guide for clinicians to rationally use these treatments and to offset any potential side-effects [9,10].

The residual lipid risk is also recognised as a potential therapeutic target for patients with ASCVD. Further reduction in LDL-c would result in decreasing future cardiovascular events [11,12]. This risk reduction was also related to the nature of ASCVD, whereby patients with high-risk features such as poly-vascular disease or previous coronary artery bypass graft (CABG) would sustain larger benefits from intensive LDL-c reduction [13,14]. Other strategies, such as the use of plaque imaging, have been proposed to maximise clinical benefits from intensive lipid-lowering treatment [3,15,16,17,18].

Beyond LDL-c, triglycerides are increasingly recognised as a risk marker and a potential target to reduce the residual risk for patients with ASCVD. Icosapent ethyl has emerged as an effective treatment for patients with hypertriglyceridemia. This review will discuss the mechanistic role of icosapent ethyl and its future role in patients with established ASCVD.

## 2. Role of Hypertriglyceridemia in Atherosclerosis

It is well-recognised that hypertriglyceridemia is linked with future adverse cardiovascular events. Early studies have demonstrated that elevated triglycerides of more than 170 mg/dL are associated with double the risk of myocardial infarction and an almost 50% increase in the risk of death [19]. Additionally, genome-wide analyses have indicated a possible causality between triglyceride-mediated pathways and coronary heart disease [20]. Moreover, mendelian randomization studies have proposed a causal relationship between triglyceride-rich lipoprotein particles and risk of ischaemic heart disease [21,22]. These studies have also suggested a possible link with low-grade inflammation [21].

Data from statin trials showed that on-treatment low triglyceride levels were associated with a reduction in adverse cardiovascular events [23]. For each 1 mmol/L decrease in triglycerides, there was an almost 10% reduction in the risk of death or recurrent myocardial infarction [23]. However, dedicated treatments to lower triglycerides have not been consistent in reducing future cardiovascular risk. Fibrates reduce triglycerides via the nuclear receptor peroxisome proliferator-activated receptor α (PPARα), with a difference in their magnitude in lowering triglycerides. Nevertheless, fibrates have not been able to demonstrate incremental clinical benefits in statin-treated patients when compared to placebo [24,25,26]. The recent Pemafibrate to Reduce Cardiovascular Outcomes by Reducing Triglycerides in Patients with Diabetes (PROMINENT) study showed that pemafibrate, a potent and selective PPARα modulator, did not reduce the composite endpoint of non-fatal myocardial infarction, ischemic stroke, coronary revascularization or cardiovascular death compared to placebo [25]. Similarly, niacin did not reduce the risk of major cardiovascular events [27,28]. The addition of 2 g of extended-release niacin and 40 mg of laropiprant in the HPS2-THRIVE study did not result in a lowering of the incidence of major cardiovascular events compared to placebo [27].

Data on marine-derived omega-3 fatty acids are conflicting [29,30]. Therefore, it is important to understand the pathophysiology and biochemical functions of omega-3 fatty acids and their effects on plasma lipoproteins and atherosclerotic plaque, including other pleotropic effects.

## 3. Omega-3 Fatty Acids

Omega-3 fatty acids are polyunsaturated fatty acids (PUFAs) that are abundant in salmon and tuna. They have more than 2 double carbons bonds, and the last one is located on the third carbon relative to the terminal methyl end of the molecule [31,32]. The effects of consuming omega-3 fatty acids (enriched in dietary fish) on reducing triglycerides and VLDL-c was recognised more than 4 decades ago [33]. The other type of PUFAs is the n-6 fatty acids, which are present in vegetable oils such as corn and soybean oils. The metabolism of n-3 and n-6 fatty acids is regulated by a similar series of enzymes, resulting in a competitive process whereby excess intake of one causes a significant reduction in the conversion of the other [32]. The downstream products of n-3 fatty acids, namely docosahexaenoic acid (DHA) and eicosapentaenoic acid (EPA), have been the focus of numerous studies aiming to reduce cardiovascular risk in patients with ASCVD [29,30,34]. On the other hand, arachidonic acid is derived from n-6 fatty acids and its eicosanoids are linked to inflammation and vasoconstriction [31].

PUFAs, including EPA and DHA, not only decrease triglycerides as a substrate but also regulate the degradation of hepatic apolipoprotein B and reduce the production of VLDL secretion [35]. In the early stages of VLDL synthesis, the ability of microsomal transfer proteins to co-translationally bind apolipoprotein B to lipids in the endoplasmic reticulum is influenced by the presence of various fatty acids. The lipidated apolipoprotein B will either acquire cholesterol ester in the hepatic cell to form intermediate-density lipoprotein (IDL) and large LDL or will fuse with a preformed lipid droplet to form VLDL particles [36]. Saturated fatty acids protect IDL and large LDL from presecretory proteolysis, while omega-3 fatty acids promote the proteolytic process, leading to a reduction in large VLDL and their subsequent product of small LDL particles [36,37]. This would explain the shift from small to large LDL particles when consuming omega-3 fatty acids and why saturated fatty acids are associated with higher level of large LDL particles [38].

**Figure 1:**
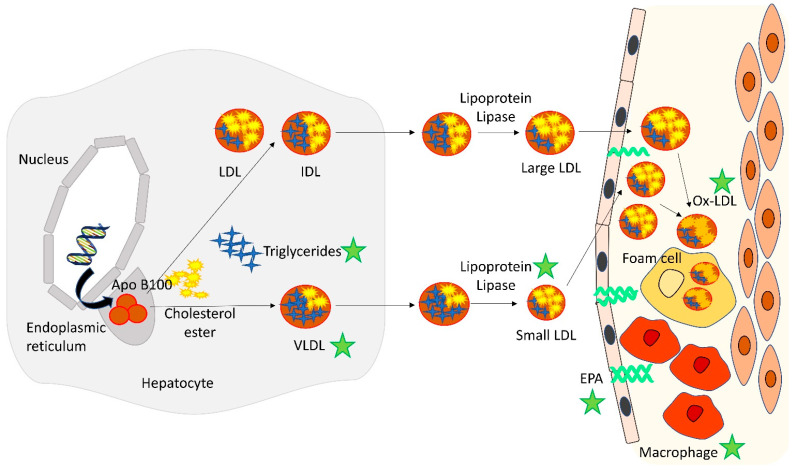
Schematic illustration of potential mechanisms of icosapent ethyl on triglycerides and atherosclerotic plaques.

PUFAs reduce triglycerides by decreasing their de novo synthesis and increasing their oxidation (Figure 1). The lipogenesis of triglycerides occurs in the hepatocytes starting from citrate, one of the substrates of the Krebs cycle. The triglyceride pathway is regulated by sterol regulatory element binding protein (SREBP)–1c, which is stimulated directly by insulin and indirectly by glucose by providing citrate via the Krebs cycle [37]. PUFAs also upregulate fatty acid oxidation via PPARα and increase its efficiency in the conversion of triglyceride-rich lipoproteins to LDL. Fibrates employ a similar mechanism in reducing triglycerides but without the ability to increase clearance of cholesterol remnant particles, resulting in no change in non-high density lipoprotein (non-HDL-c) level [39]. Although pemafibrate caused a 25% reduction in triglycerides, VLDL-c and remnant cholesterol, there was a more than 10% increase in LDL-c and an almost 5% increase in apolipoprotein B level compared to placebo [25]. Therefore, for triglyceride-lowering treatment to be effective in reducing cardiovascular risk, it may be dependent on its ability to reduce apolipoprotein B particles and not merely converting triglyceride-rich lipoprotein to LDL. Finally, PUFAs have opposing effects on nuclear receptors that regulate cholesterol clearance via the bile acid and inhibiting the activation of adenosine triphosphate–binding cassette transporter (ABC) A1 that promotes efflux of cholesterol into HDL [39].

## 4. Eicosapentaenoic Acid (EPA) and Docosahexaenoic Acid (DHA)

EPA and DHA are omega-3 PUFAs, but they differ in their effects on lipoprotein particles, oxidative features, inflammatory biomarkers, cellular membrane structure and tissue distribution. There is a divergent influence on LDL between EPA and DHA, whereby the former has neutral effect compared to a marginal increase in LDL of 5–10 mg/dL with DHA [40,41,42]. This may be related to the more potent inhibitory effect of EPA on diacylglycerol acyl transferase compared to DHA, resulting in a greater effect on triglyceride synthesis [43,44]. Additionally, EPA better stimulates lipoprotein lipase, enhancing triglycerides clearance [45,46]. Furthermore, animal models suggest that DHA may down-regulate the LDL receptor, increasing levels of LDL-c [47]. DHA increases HDL and causes a greater reduction in triglycerides compared to EPA [41]. On the other hand, the magnitude of reduction in non-HDL and apolipoprotein B is larger in EPA-containing treatments compared to DHA-containing treatments [40].

Beyond their impact on lipid profiles, omega-3 fatty acids are well recognised to be incorporated within the atherosclerotic plaque. This role may contribute to their protective effect on plaque inflammation and stability [48,49,50]. In explanted carotid atherosclerotic plaque specimens, patients treated with fish oil (omega-3 fatty acids) had fewer thin fibrous cap plaques and signs of inflammation compared to those subjected to sunflower oil or placebo [49]. Intriguingly, EPA was more readily included in atherosclerotic plaques, although it was simultaneously administered with DHA and was translated into a smaller number of foam cells and T-cells, i.e., less inflammatory [48]. In vivo assessment of coronary plaques using integrated backscatter ultrasound virtual histology revealed that a lower serum content of EPA was associated with high-risk plaque features such as large lipid burden and small fibrous volume [50]. Low EPA, but not DHA, was an independent predictor for lipid-rich plaques [50].

EPA was early recognised as an inhibitor of platelet aggregation. Various mechanisms were postulated, including the formation of thromboxane A3, production of prostacyclin (PCI3) and shift from producing thromboxane A2 [51]. It was also proposed that EPA works synergistically with aspirin in reducing the risk of thrombosis [52].

Longitudinal studies have validated the differential biochemical and biological effects of omega-3 fatty acids on cardiovascular outcomes. Unlike DHA, low EPA levels were associated with increased mortality in patients with acute myocardial infarction [53]. Similarly, low EPA:AA ratios were associated with increased risk of coronary heart disease [54]. This relationship was more evident in patients with elevated high-sensitivity CRP [54]. Such correlation was not present with low DHA:AA ratios [54]. Collectively, these differences would inevitably explain the inconsistency in the outcomes of clinical studies assessing the role of omega-3 fatty acids on cardiovascular outcomes [55].

## 5. Clinical Outcomes Studies- STRENGTH and REDUCE-IT

Numerous studies have assessed the role of omega-3 fatty acids on cardiovascular outcomes (Table 1). The Gruppo Italiano per lo Studio della Sopravvivenza nell’ Infarto miocardico (GISSI)-Prevenzione trial included 11,324 patients with recent myocardial infarction (≤3 months) and were randomly assigned supplements of omega-3 PUFAs or vitamin E in a two-by-two factorial design [56]. Omega-3 PUFAs were given as a mixture of EPA (850 mg) and DHA (882 mg). After 3.5 years follow up, there was 11% risk reduction in the composite of cardiovascular death, non-fatal myocardial infarction or stroke [relative risk (RR) 0.89, 95% confidence interval (CI) (0.80–1.01)], which was more evident when the analysis was restricted to the use of omega-3 PUFAs (4-way analysis) [RR 0.80, 95% CI (0.68–0.95)] [56]. Notably, the baseline LDL-c and triglycerides were 137 mg/dL and 162 mg/dL, respectively [56]. Only 5% of patients received cholesterol-lowering treatment at baseline, which rose to 45% at the end of the study follow up [56]. The same group assessed the role of omega-3 PUFAs using the same dose in 6975 patients with chronic heart failure. There was a significant reduction in the incidence of all-cause mortality after 3.9 years of consuming omega-3 PUFAs [adjusted hazard ratio [HR] 0.91, 95% CI (0.833–0.998), *p* = 0.041] [57]. This reduction was mainly driven by worsening heart failure or presumed arrhythmic deaths and was more evident in patients with diabetes. Intriguingly, there was no change in total, HDL or LDL cholesterol, highlighting the pleotropic effect of omega-3 PUFAs as anti-arrhythmic and membrane-stabilising drugs [57].

The JAPAN EPA Lipid Intervention Study (JELIS) randomised 18,645 patients with a total cholesterol of 6.5 mmol/L or greater to receive either a combination of statin and EPA (1800 mg) or statin alone [58]. Up to 1 in 7 of patients had history of coronary artery disease and their mean triglyceride level was 153 mg/dL [58]. After 5 years follow up, major coronary events (defined as the composite endpoint of sudden cardiac death, fatal and non-fatal myocardial infarction, and other non-fatal events, including unstable angina pectoris, angioplasty, stenting, or coronary artery bypass grafting) were significantly reduced in patients receiving the combination of EPA and statin compared to statin alone [2.8% versus 3.5%, HR 0.81, 95% CI (0.69–0.95), *p* = 0.011] [58].

Four large randomised clinical trials, each recruiting more than 12,000 participants, assessed the role of omega-3 PUFAs in reducing future cardiovascular risk in relatively lower risk cohorts [61,62,64,65]. They included patients with diabetes (without previous ASCVD) at risk of cardiovascular disease or without previous history of cardiovascular disease (primary prevention) with follow up of more than 5 years [61,62,64,65]. The primary endpoint of cardiovascular death, myocardial infarction, stroke or revascularisation was comparable between patients receiving omega-3 PUFAs and placebo [61,62,64,65]. It is important to highlight that the received dose of omega-3 fatty acid was relatively modest (less than 1 g per day) with a mixture of EPA and DHA at a ratio of 1–1.2:1 [61,62,64,65].

The Reduction of Cardiovascular Events with Icosapent Ethyl–Intervention Trial (REDUCE-IT) was a landmark trial that established the protective cardiovascular effect of omega-3 fatty acids [29]. It randomly assigned 8179 statin-treated patients with triglyceride levels of 135 to 499 mg/dL and established cardiovascular disease (70%) or with diabetes and other risk factors (30%) to receive a highly purified dose of EPA (2 g of icosapent ethyl twice daily; total daily dose of 4 g) [29]. After a median follow up of 4.9 years, the primary endpoint (defined as a composite of cardiovascular death, nonfatal myocardial infarction, nonfatal stroke, coronary revascularization or unstable angina) was significantly reduced in the icosapent ethyl group compared with placebo [17.2% vs. 22.0%, HR 0.75, 95% CI (0.68 to 0.83), *p* < 0.001] [29]. These benefits were more marked in patients with a history of myocardial infarction of less than one year. There was a reduction in cardiovascular death with icosapent ethyl treatment [4.3% vs. 5.2%, HR 0.80, 95% CI (0.66 to 0.98), *p* = 0.03] [29]. Interestingly, the clinical benefits with icosapent ethyl were not proportionate to the attained triglyceride levels, and lowering of major cardiovascular events was evident in patients with triglyceride levels of ≥150 or <150 mg/dL. On the other hand, the on-treatment EPA level correlated with the accrued clinical benefits of icosapent ethyl [67]. Moreover, the Kaplan-Meier curves started to diverge after 18 months’ treatment with icosapent ethyl, suggesting a lipoprotein-related mechanism reduced cardiovascular events. The reduction in atherogenic lipoprotein particles coupled with incorporation of EPA within cellular membranes would stabilise atherosclerotic plaques by changing their composition and reducing their inflammatory cellular content. Early initiation of EPA demonstrated a significant reduction in adverse clinical events in patients with acute coronary syndrome [68]. In this relatively small study of 241 patients, commencing EPA during hospitalisation resulted in an almost 60% reduction in cardiovascular death, nonfatal stroke, nonfatal myocardial infarction and revascularization [68].

In contrast, the Long-Term Outcomes Study to Assess Statin Residual Risk with Epanova in High Cardiovascular Risk Patients with Hypertriglyceridemia (STRENGTH) trial was prematurely halted after an interim analysis indicating the low probability of benefits from omega-3 PUFAs compared to placebo [30]. The STRENGTH trial randomly assigned 13,078 statin-treated patients with triglyceride levels of 180–500 mg/dL to daily treatment of omega-3 capsules containing 4 g of an EPA/DHA mixture or a matching corn oil placebo. Only 55% of the patients had established ASCVD and the study allowed the inclusion of high-risk primary prevention patients. The primary endpoint (defined as the composite of cardiovascular death, nonfatal myocardial infarction, nonfatal stroke, coronary revascularization or unstable angina requiring hospitalization) was not different between the two groups [12% vs. 12.2%, HR 0.99, 95% CI (0.90–10.9), *p* = 0.84] [30].

Recently, the Randomized trial for Evaluation in Secondary Prevention Efficacy of Combination Therapy—Statin and EPA (RESPECT-EPA) was presented at the American Heart Association in 2022 [66]. The study randomised 2506 statin-treated patients with a median LDL-c of 81 mg/dL and a median triglyceride level of 119 mg/dL to icosapent ethyl 1800 mg/day or no additional treatment. The targeted cohort was patients with stable CAD and an EPA:AA ratio below 0.4. The primary outcome of cardiovascular death, myocardial infarction, stroke, unstable angina requiring hospitalization, and revascularization, occurred in 10.9% in the icosapent ethyl group vs. 14.9% in the control group (*p* = 0.055). A post hoc analysis that included patients with a large between-group increase in EPA level revealed a significant reduction in the primary endpoint with icosapent ethyl [HR 0.73, 95% CI (0.55–0.95)].

## 6. The Divergence in the Results of Clinical Outcomes Data

Clinical outcome studies differ in their included population, the dose and the ‘purity’ of the omega-3 fatty acids used, and likely explain the inconsistency in their reported outcomes. The striking difference was very evident when comparing the results of the STRENGTH and REDUCE-IT trials. Whilst there are some variations in the proportion of patients with established ASCVD between the two studies, this is unlikely on its own to explain such a difference in their results. The administered dose of EPA combined with its purity would play a bigger role when comparing both studies. The baseline EPA in the REDUCE-IT was 26 µg/mL, which rose to 144 µg/mL (an almost 400% increase) compared to an attained EPA level of 89 µg/mL in the STRENGTH trial. Interestingly, the STRENGTH trial did not demonstrate any association between on-treatment EPA level and subsequent cardiovascular outcomes, including patients who achieved an increase in EPA level of >400% [30]. The on-treatment EPA level in the JELIS trial was 97 µg/mL, suggestive of a possible threshold of plasma EPA level to exert clinical benefits [67]. A recent meta-analysis of 197,270 patients suggested a 7% proportionate reduction in cardiovascular outcomes for each 1 g daily administration of EPA [34]. Such association was not present with DHA. [34]. In fact, the presence of DHA may modulate the effect of EPA-containing treatment and may exhibit antagonist effects when integrated as phospholipids within membrane structures [69].

The controversy related to the use of mineral oil as a placebo in the REDUCE-IT trial may have also magnified the clinical benefits with icosapent ethyl. Compared to baseline, there was a median increase of 10.9% in oxidised LDL-c, 18.5% for lipoprotein-associated phospholipase A2 and 21.9% for high-sensitivity C-reactive protein [70]. Nonetheless, the absolute difference in these biomarkers including LDL-c was relatively modest and unlikely to explain the reported clinical benefits in the REDUCE-IT trial. Moreover, the reduction in cardiovascular events in response to icosapent ethyl was not influenced by the direction of change in LDL-c or hs-CRP in the placebo arm [67,71]. Furthermore, the US food and Drug Administration concluded that only a small fraction, if any, of the difference in clinical outcomes in the REDUCE-IT trial could be explained by the use of mineral oil as a placebo [67]. Finally, in vitro model did not reveal any oxidation effect of atherogenic lipoprotein particles when subjected to mineral or corn oils [72].

The recent RESPECT-EPA study has highlighted probable clinical benefits when using icosapent ethyl, although it marginally missed the statistical significance threshold. Despite its relatively small sample size, it included patients who were most likely to benefit from omega-3 PUFA treatment. This was borne out in the subgroup analysis that included patients with a large increase in their attained EPA. Importantly, the RESPECT-EPA did not assign any placebo oil to the control arm, supporting the notion that the clinical benefits in the REDUCE-IT trial was not merely derived from the presence of mineral oil, and the latter had a small and negligible effect on the study outcome.

## 7. Changes in Atherosclerotic Plaque in Response to Omega-3 PUFAs

The relationship between omega-3 fatty acids and plaque characteristics was proposed more than a decade ago. Serum levels of omega-3 fatty acids, particularly EPA, were demonstrated to be inversely correlated with the percentage of plaque lipid volume, quantified using integrated backscatter intra-vascular ultrasound in patients undergoing coronary angiography [50]. Low EPA levels were associated with a more than two-fold risk of identifying lipid-rich plaques [50].

Both invasive and non-invasive imaging modalities were used to assess the effect of omega-3 fatty acids on atherosclerotic plaque burden and characteristics (Table 2) [73]. The Effect of Vascepa on Improving Coronary Atherosclerosis in People With High Triglycerides Taking Statin Therapy (EVAPORATE) study randomised 80 patients to 4 g daily of icosapent ethyl or mineral oil [74]. The use of icosapent ethyl resulted in a significant reduction in plaque volume after 18 months as quantified on coronary computed tomography (CT). Additionally, there was a significant reduction in low attenuation, fibrofatty, fibrous and non-calcified plaque volume [74]. Calcification was numerically lower with icosapent ethyl, but this did not reach statistical significance [74]. Similar findings were reported from 210 patients with acute coronary syndrome who underwent serial coronary CT and demonstrated that high-dose EPA was the least associated with plaque progression, including the absence of high-risk features [75]. Such difference in plaque volume or composition was not present in patients with stable coronary artery disease [76].

The use of omega-3 fatty acids was also associated with plaque stabilisation effects using intravascular ultrasound and optical coherent tomography (OCT). These effects included a reduction in lipid volume and an increase in fibrous-cap thickness [77,78,79]. Additionally, macrophage and inflammatory cytokines were less frequently detected in patients subjected to omega-3 fatty acids compared to control [79,80,81]. These anti-inflammatory properties of EPA also include reversing endothelial dysfunction when exposed to small dense LDL, increasing nitric oxide release (a potent inhibitor of platelet aggregation), decreasing lipoprotein associated phospholipase A2, and oxidation of LDL-c [67].

**Table 2:** Summary of clinical imaging trials assessing the role of omega-3 fatty acids on atherosclerotic plaque.

Study Name	Study Size	Targeted Cohort	EPA/DHA Dose	Imaging Modality	Imaging Interval	Change in Plaque Imaging
Nishio et al. (2014) [79]	30	CAD undergoing PCI	1800:0	Optical coherent tomography	9 months	A greater increase in fibrous-cap thickness, with a greater decrease in lipid arc, lipid length and macrophage accumulation was detected in the EPA group
Yamano et al. (2015) [78]	46	Acute coronary syndrome	1800:0	Optical coherent tomography	8 months	The lipid arc did not change, but the relative change in the fibrous cap thickness was significantly greater in the EPA group than in the control group
Ahn et al. (2016) [82]	74	CAD undergoing PCI	1395:1125	Intravascular ultrasound	12 months	No significant differences were observed in atheroma volume
Niki et al. (2016) [80]	95	CAD undergoing PCI	1800:0	Integrated backscatter intravascular ultrasound	6 months	A significant reduction in lipid volume and a significant increase in fibrous volume were evident in the PUFAs group
CHERRY [77]	193	CAD undergoing PCI	1800:0	Integrated backscatter intravascular ultrasound	6–8 months	Greater reduction in total atheroma and lipid volume with combination of pitavastatin and PUFAs compared to pitavastatin only
Alfaddagh et al. (2017) [76]	285	Stable CAD	1860:1500	Coronary computed tomographic angiography	30 months	No difference in non-calcified plaque volume between the groups
EVAPORATE (2020) [74]	80	≥20% angiographic stenosis on	4000:0	Multidetector computed tomography	18 months	A significant reduction in low-attenuation plaque volume with PUFAs compared to the control group
Kita et al. (2020) [83]	130	Acute coronary syndrome	1800:0	Optical coherent tomography	8 months	PUFA therapy in addition to strong statin therapy did not significantly increase FCT in plaques compared with strong statin therapy alone, but significantly increased FCT in patients with thinner FCT
Sugizaki et al. (2020) [81]	42	CAD undergoing optical coherent tomography	1800:0	Optical coherent tomography	12 months	A significantly greater decrease in the lipid index and macrophage grade in patients receiving rosuvastatin 10 mg and EPA compared to 2.5 mg rosuvastatin only
Motoyama et al. (2022) [75]	210	Acute coronary syndrome	1800:0	Coronary computed tomographic angiography	24 months	5.6% had plaque progression in the EPA group compared to 20.3% in the control one

CAD: Coronary artery disease; FCT: Fibrous cap thickness; PCI: Percutaneous coronary intervention.

## 8. Omega-3 Fatty Acids Safety Profile

Omega-3 PUFAs are considered safe with no reported fatal side effects. The rate of gastrointestinal adverse events has been reported in up to one-third of patients receiving omega-3 fatty acids, although this was comparable to the placebo [29]. In the STRENGTH study, the rate of gastrointestinal side effects was almost doubled with EPA/DHA treatment compared to corn oil [30].

The most serious side effect is new-onset atrial fibrillation. The REDUCE-IT trial reported a significant increase in the rate of atrial fibrillation in the icosapent ethyl group compared to the placebo group (5.3% vs. 3.9%) [29]. Similarly, the RESPECT-EPA study highlighted an almost two-fold increase in new atrial fibrillation (3.1% vs. 1.6%; *p* = 0.017). Importantly, the hospitalization for atrial fibrillation was also significantly higher in the icosapent ethyl group in the REDUCE-IT trial (3.1% vs. 2.1%, *p* = 0.004) [29]. A recent meta-analysis of 81,210 patients from 7 trials revealed a 25% increased risk of atrial fibrillation with omega-3 fatty acids [HR 1.25, 95%CI (1.07–1.46), *p* = 0.013] [84]. There was a differential effect according to the dose of omega-3 fatty acids with an almost 50% increase in the risk of atrial fibrillation in patients receiving >1 g per day of omega-3 fatty acids [84]. Interestingly, data from the Multi-Ethnic Study of Atherosclerosis suggested that higher DHA levels (but not EPA or EPA + DHA) are associated with fewer atrial fibrillation events [85].

Although the overall rate of bleeding events was numerically higher in the icosapent ethyl group in the REDUCE-IT trial compared to placebo (2.7% vs. 2.1%), this difference did not reach statistical significance (*p* = 0.06). More importantly, the RESPECT-EPA and STRENGTH trials reported comparable bleeding events with placebo. Furthermore, the incidence of serious bleeding events (haemorrhagic stroke or central nervous system bleeding events) was rare (<0.5%).

In conclusion, recent clinical and imaging studies have demonstrated the protective effects of highly purified omega-3 fatty acids in reducing residual cardiovascular risk in statin-treated patients. The inconsistency in some clinical outcome data is likely related to the type of experimented omega-3 PUFA and the targeted cohort. The disconnection between cardiovascular risk reduction with omega-3 PUFAs and triglyceride levels would urge better identification of patients who are likely to benefit from this emerging treatment.

## Figures and Tables

**Table 1:** Summary of landmark clinical trials assessing the role of omega-3 fatty acids on cardiovascular outcomes.

Study Name	Study Size	Targeted Cohort	LDL-c Level	TG Level	Statin-Treated%	EPA/DHA Dose	Primary Endpoint	Follow Up	Relative Risk
GISSI (1999) [56]	11,324	Post MI (≤3 months)	137 mg/dL	162 mg/dL	45% *	850:882	death, non-fatal MI, or stroke	3.5 years	0.90 (0.82–0·99)
JELIS (2007) [58]	18,645	Total chol ≥ 6.5 mmol/L	182 mg/dL	154 mg/dL	98%	1800:0	SCD, fatal and non-fatal MI, unstable angina, or revasc	5 years	0.81 (0.69–0.95)
GISSI-HF (2008) [57]	6975	Chronic heart failure	- ^†^	126 mg/dL	23%	850:882	Death or re-admission to hospital for CV reason	3.9 years	0.92 (0.84–0.99)
Alpha Omega (2010) [59]	4837	Post MI (up to 10 years)	100 mg/dL	146 mg/dL	86%	226:150	Fatal, non-fatal CV disease, or revasc	40 months	1.01 (0.87–11.17)
SU.FUL. OM3 (2010) [60]	2501	History of MI, unstable angina, or ischaemic stroke	104 mg/dL	106 mg/dL	85%	400:200	CV death, non-fatal MI, or stroke	4.7 years	1.08 (0.79–1.47)
ORIGIN (2012) [61]	12,536	Diabetic, or IGT with high risk for CV event	112 mg/dL	141 mg/dL	54%	465:375	CV death	6.2 years	0.98 (0.87–1.10)
R&P (2013) [62]	12,513	Multiple CV risk factors or ASCVD (but not MI)	132	150	41%	500:500	Death, non-fatal MI, or stroke	5 yeas	0.97 (0.88–1.08)
AREDs (2014) [63]	4203	Inter- or advanced ARMD	-	-	44%	650:350	CV death, non-fatal MI, or stroke	4.8 years	0.95 (0.78–1.17)
ASCEND (2018) [64]	15,480	Diabetic without ASCVD	113 ^∫∫^	-	76%	460:380	CV death, non-fatal MI, or stroke	7.4 years	0.97 (0.87–1.08)
VITAL (2019) [65]	25,871	Men ≥ 50 or women ≥ 55 years	-	-	37%	460:380	CV death, non-fatal MI, or stroke	5.3 years	0.92 (0.80–1.06)
REDUCE-IT (2019) [29]	8179	Est CV disease or diabetes and other risk factors	75 mg/dL	216 mg/dL	100%	4000:0	CV death, non-fatal MI, stroke, revasc, or unstable angina	4.9 years	0.75 (0.68–0.83)
STRENGTH (2020) [30]	13,078	High CV risk	75 mg/dL	240 mg/dL	100%	2200:800	CV death, non-fatal MI, stroke, revasc, or unstable angina	42 months	0.99 (0.90–1.09)
RESPECT-EPA (2023) [66]	2506	CAD with EPA: AA ratio < 0.4	81 mg/dL	119 mg/dL	100%	1800:0	CV death, non-fatal MI, stroke, revasc, or unstable angina	At least 4 years	0.78 (0.61–1.00)

* By the end of the study, ^†^ 55% of included patients had total cholesterol > 188 mg/dL, ^∫∫^ Measurement of non-HDL-c. ARMD: age-related macular degeneration; CAD: coronary artery disease; Chol: cholesterol; CV: cardiovascular; Est: established; IGT: impaired glucose tolerance; Inter: intermediate; MI: myocardial infarction; Revasc: revascularisation; SCD: sudden cardiac death; TG: triglycerides.

## Data Availability

Not applicable.

## References

[1] Visseren F.L.J., Mach F., Smulders Y.M., Carballo D., Koskinas K.C., Back M., Benetos A., Biffi A., Boavida J.M., Capodanno D. (2021). 2021 ESC Guidelines on cardiovascular disease prevention in clinical practice. Eur. Heart J..

[2] Cannon C.P., Blazing M.A., Giugliano R.P., McCagg A., White J.A., Theroux P., Darius H., Lewis B.S., Ophuis T.O., Jukema J.W. (2015). Ezetimibe Added to Statin Therapy after Acute Coronary Syndromes. N. Engl. J. Med..

[3] Alkhalil M. (2021). Mechanistic Insights to Target Atherosclerosis Residual Risk. Curr. Probl. Cardiol..

[4] Ridker P.M., Everett B.M., Thuren T., MacFadyen J.G., Chang W.H., Ballantyne C., Fonseca F., Nicolau J., Koenig W., Anker S.D. (2017). Antiinflammatory Therapy with Canakinumab for Atherosclerotic Disease. N. Engl. J. Med..

[5] Wallentin L., Becker R.C., Budaj A., Cannon C.P., Emanuelsson H., Held C., Horrow J., Husted S., James S., Katus H. (2009). Ticagrelor versus clopidogrel in patients with acute coronary syndromes. N. Engl. J. Med..

[6] Wiviott S.D., Braunwald E., McCabe C.H., Montalescot G., Ruzyllo W., Gottlieb S., Neumann F.J., Ardissino D., De Servi S., Murphy S.A. (2007). Prasugrel versus clopidogrel in patients with acute coronary syndromes. N. Engl. J. Med..

[7] Eikelboom J.W., Connolly S.J., Bosch J., Dagenais G.R., Hart R.G., Shestakovska O., Diaz R., Alings M., Lonn E.M., Anand M.D. (2017). Rivaroxaban with or without Aspirin in Stable Cardiovascular Disease. N. Engl. J. Med..

[8] Tardif J.C., Kouz S., Waters D.D., Bertrand O.F., Diaz R., Maggioni A.P., Pinto F.J., Ibrahim R., Gamra H., Kiwan G.S. (2019). Efficacy and Safety of Low-Dose Colchicine after Myocardial Infarction. N. Engl. J. Med..

[9] Kuzemczak M., Ibrahem A., Alkhalil M. (2021). Colchicine in Patients with Coronary Artery Disease with or Without Diabetes Mellitus: A Meta-analysis of Randomized Clinical Trials. Clin. Drug Investig..

[10] Ridker P.M., MacFadyen J.G., Everett B.M., Libby P., Thuren T., Glynn R.J., CANTOS Trial Group (2018). Relationship of C-reactive protein reduction to cardiovascular event reduction following treatment with canakinumab: A secondary analysis from the CANTOS randomised controlled trial. Lancet.

[11] Schwartz G.G., Steg P.G., Szarek M., Bhatt D.L., Bittner V.A., Diaz R., Edelberg J.M., Goodman S.G., Hanotin C., Harrington R.A. (2018). Alirocumab and Cardiovascular Outcomes after Acute Coronary Syndrome. N. Engl. J. Med..

[12] Sabatine M.S., Giugliano R.P., Keech A.C., Honarpour N., Wiviott S.D., Murphy S.A., Kuder J.F., Wang H., Liu T., Wasserman S.M. (2017). Evolocumab and Clinical Outcomes in Patients with Cardiovascular Disease. N. Engl. J. Med..

[13] Alkhalil M. (2020). Effects of intensive lipid-lowering therapy on mortality after coronary bypass surgery: A meta-analysis of 7 randomised trials. Atherosclerosis.

[14] Alkhalil M., Kuzemczak M., Whitehead N., Kavvouras C., Dzavik V. (2021). Meta-Analysis of Intensive Lipid-Lowering Therapy in Patients with Polyvascular Disease. J. Am. Heart Assoc..

[15] Alkhalil M., Biasiolli L., Chai J.T., Galassi F., Li L., Darby C., Halliday A., Hands L., Magee T., Perkins J. (2017). Quantification of carotid plaque lipid content with magnetic resonance T2 mapping in patients undergoing carotid endarterectomy. PLoS ONE.

[16] Alkhalil M., Chai J.T., Choudhury R.P. (2017). Plaque imaging to refine indications for emerging lipid-lowering drugs. Eur. Heart J. Cardiovasc. Pharmacother..

[17] Alkhalil M., Biasiolli L., Akbar N., Galassi F., Chai J.T., Robson M.D., Choudhury R.P. (2018). T2 mapping MRI technique quantifies carotid plaque lipid, and its depletion after statin initiation, following acute myocardial infarction. Atherosclerosis.

[18] Alkhalil M. (2019). Proprotein Convertase Subtilisin/Kexin Type 9 (PCSK9) Inhibitors, Reality or Dream in Managing Patients with Cardiovascular Disease. Curr. Drug Metab..

[19] Nordestgaard B.G., Benn M., Schnohr P., Tybjaerg-Hansen A. (2007). Nonfasting triglycerides and risk of myocardial infarction, ischemic heart disease, and death in men and women. JAMA.

[20] Sarwar N., Sandhu M.S., Ricketts S.L., Butterworth A.S., Di Angelantonio E., Boekholdt S.M., Ouwehand W., Watkins H., Triglyceride Coronary Disease Genetics Consortium, Emerging Risk Factors Collaboration (2010). Triglyceride-mediated pathways and coronary disease: Collaborative analysis of 101 studies. Lancet.

[21] Varbo A., Benn M., Tybjaerg-Hansen A., Nordestgaard B.G. (2013). Elevated remnant cholesterol causes both low-grade inflammation and ischemic heart disease, whereas elevated low-density lipoprotein cholesterol causes ischemic heart disease without inflammation. Circulation.

[22] Varbo A., Benn M., Tybjaerg-Hansen A., Jorgensen A.B., Frikke-Schmidt R., Nordestgaard B.G. (2013). Remnant cholesterol as a causal risk factor for ischemic heart disease. J. Am. Coll. Cardiol..

[23] Miller M., Cannon C.P., Murphy S.A., Qin J., Ray K.K., Braunwald E. (2008). PROVE IT-TIMI 22 Investigators. Impact of triglyceride levels beyond low-density lipoprotein cholesterol after acute coronary syndrome in the PROVE IT-TIMI 22 trial. J. Am. Coll. Cardiol..

[24] Arai H., Yamashita S., Yokote K., Araki E., Suganami H., Ishibashi S., K-877 Study Group (2017). Efficacy and safety of K-877, a novel selective peroxisome proliferator-activated receptor alpha modulator (SPPARMalpha), in combination with statin treatment: Two randomised, double-blind, placebo-controlled clinical trials in patients with dyslipidaemia. Atherosclerosis.

[25] Das Pradhan A., Glynn R.J., Fruchart J.C., MacFadyen J.G., Zaharris E.S., Everett B.M., Campbell S.E., Oshima R., Amarenco P., Blom D.J. (2022). Triglyceride Lowering with Pemafibrate to Reduce Cardiovascular Risk. N. Engl. J. Med..

[26] Ginsberg H.N., Elam M.B., Lovato L.C., Crouse J.R., Leiter L.A., Linz P., Friedewald W.T., Buse J.B., Gerstein H.C., ACCORD Study Group (2010). Effects of combination lipid therapy in type 2 diabetes mellitus. N. Engl. J. Med..

[27] Landray M.J., Haynes R., Hopewell J.C., Parish S., Aung T., Tomson J., Wallendszus K., Craig M., Jiang L., HPS2-Thrive Collaborative Group (2014). Effects of extended-release niacin with laropiprant in high-risk patients. N. Engl. J. Med..

[28] Boden W.E., Probstfield J.L., Anderson T., Chaitman B.R., Desvignes-Nickens P., Koprowicz K., McBride R., Teo K., Weintraub W., Aim-High Investigators (2011). Niacin in patients with low HDL cholesterol levels receiving intensive statin therapy. N. Engl. J. Med..

[29] Bhatt D.L., Steg P.G., Miller M., Brinton E.A., Jacobson T.A., Ketchum S.B., Doyle R.T., Juliano R.A., Jiao L., Granowitz C. (2019). Cardiovascular Risk Reduction with Icosapent Ethyl for Hypertriglyceridemia. N. Engl. J. Med..

[30] Nicholls S.J., Lincoff A.M., Garcia M., Bash D., Ballantyne C.M., Barter P.J., Davidson M.H., Kastelein J.J.P., Koenig W., McGuire D.K. (2020). Effect of High-Dose Omega-3 Fatty Acids vs Corn Oil on Major Adverse Cardiovascular Events in Patients at High Cardiovascular Risk: The STRENGTH Randomized Clinical Trial. JAMA.

[31] Schmitz G., Ecker J. (2008). The opposing effects of n-3 and n-6 fatty acids. Prog. Lipid Res..

[32] Grundy S.M. (2003). N-3 fatty acids: Priority for post-myocardial infarction clinical trials. Circulation.

[33] Phillipson B.E., Rothrock D.W., Connor W.E., Harris W.S., Illingworth D.R. (1985). Reduction of plasma lipids, lipoproteins, and apoproteins by dietary fish oils in patients with hypertriglyceridemia. N. Engl. J. Med..

[34] Marston N.A., Giugliano R.P., Im K., Silverman M.G., O’Donoghue M.L., Wiviott S.D., Ference B.A., Sabatine M.S. (2019). Association between Triglyceride Lowering and Reduction of Cardiovascular Risk Across Multiple Lipid-Lowering Therapeutic Classes: A Systematic Review and Meta-Regression Analysis of Randomized Controlled Trials. Circulation.

[35] Pan M., Cederbaum A.I., Zhang Y.L., Ginsberg H.N., Williams K.J., Fisher E.A. (2004). Lipid peroxidation and oxidant stress regulate hepatic apolipoprotein B degradation and VLDL production. J. Clin. Investig..

[36] Krauss R.M. (2004). Hold the antioxidants and improve plasma lipids?. J. Clin. Investig..

[37] Davidson M.H. (2006). Mechanisms for the hypotriglyceridemic effect of marine omega-3 fatty acids. Am. J. Cardiol..

[38] Ballantyne C.M., Braeckman R.A., Bays H.E., Kastelein J.J., Otvos J.D., Stirtan W.G., Doyle R.T., Soni P.N., Juliano R.A. (2015). Effects of icosapent ethyl on lipoprotein particle concentration and size in statin-treated patients with persistent high triglycerides (the ANCHOR Study). J. Clin. Lipidol..

[39] Virani S.S. (2022). The Fibrates Story—A Tepid End to a PROMINENT Drug. N. Engl. J. Med..

[40] Skulas-Ray A.C., Wilson P.W.F., Harris W.S., Brinton E.A., Kris-Etherton P.M., Richter C.K., Jacobson T.A., Engler M.B., Miller M., Robinson J.G. (2019). Omega-3 Fatty Acids for the Management of Hypertriglyceridemia: A Science Advisory From the American Heart Association. Circulation.

[41] Wei M.Y., Jacobson T.A. (2011). Effects of eicosapentaenoic acid versus docosahexaenoic acid on serum lipids: A systematic review and meta-analysis. Curr. Atheroscler Rep..

[42] Toth P.P., Chapman M.J., Parhofer K.G., Nelson J. (2022). Differentiating EPA from EPA/DHA in cardiovascular risk reduction. Am. Heart J. Plus Cardiol. Res. Pract..

[43] Berge R.K., Madsen L., Vaagenes H., Tronstad K.J., Gottlicher M., Rustan A.C. (1999). In contrast with docosahexaenoic acid, eicosapentaenoic acid and hypolipidaemic derivatives decrease hepatic synthesis and secretion of triacylglycerol by decreased diacylglycerol acyltransferase activity and stimulation of fatty acid oxidation. Biochem J..

[44] Madsen L., Rustan A.C., Vaagenes H., Berge K., Dyroy E., Berge R.K. (1999). Eicosapentaenoic and docosahexaenoic acid affect mitochondrial and peroxisomal fatty acid oxidation in relation to substrate preference. Lipids.

[45] Forman B.M., Chen J., Evans R.M. (1997). Hypolipidemic drugs, polyunsaturated fatty acids, and eicosanoids are ligands for peroxisome proliferator-activated receptors alpha and delta. Proc. Natl. Acad. Sci. USA.

[46] Jump D.B., Botolin D., Wang Y., Xu J., Demeure O., Christian B. (2008). Docosahexaenoic acid (DHA) and hepatic gene transcription. Chem. Phys. Lipids..

[47] Ishida T., Ohta M., Nakakuki M., Kami H., Uchiyama R., Kawano H., Notsu T., Imada K., Shimano H. (2013). Distinct regulation of plasma LDL cholesterol by eicosapentaenoic acid and docosahexaenoic acid in high fat diet-fed hamsters: Participation of cholesterol ester transfer protein and LDL receptor. Prostaglandins Leukot Essent Fat. Acids.

[48] Cawood A.L., Ding R., Napper F.L., Young R.H., Williams J.A., Ward M.J., Gudmundsen O., Vige R., Payne S.P., Ye S. (2010). Eicosapentaenoic acid (EPA) from highly concentrated n-3 fatty acid ethyl esters is incorporated into advanced atherosclerotic plaques and higher plaque EPA is associated with decreased plaque inflammation and increased stability. Atherosclerosis.

[49] Thies F., Garry J.M., Yaqoob P., Rerkasem K., Williams J., Shearman C.P., Gallagher P.J., Calder P.C., Grimble R.F. (2003). Association of n-3 polyunsaturated fatty acids with stability of atherosclerotic plaques: A randomised controlled trial. Lancet.

[50] Amano T., Matsubara T., Uetani T., Kato M., Kato B., Yoshida T., Harada K., Kumagai S., Kunimura A., Shinbo Y. (2011). Impact of omega-3 polyunsaturated fatty acids on coronary plaque instability: An integrated backscatter intravascular ultrasound study. Atherosclerosis.

[51] Dyerberg J., Bang H.O., Stoffersen E., Moncada S., Vane J.R. (1978). Eicosapentaenoic acid and prevention of thrombosis and atherosclerosis?. Lancet.

[52] Gong Y., Lin M., Piao L., Li X., Yang F., Zhang J., Xiao B., Zhang Q., Song W.L., Yin H. (2015). Aspirin enhances protective effect of fish oil against thrombosis and injury-induced vascular remodelling. Br. J. Pharmacol..

[53] Lee S.H., Shin M.J., Kim J.S., Ko Y.G., Kang S.M., Choi D., Jang Y., Chung N., Shim W.H., Cho S.Y. (2009). Blood eicosapentaenoic acid and docosahexaenoic acid as predictors of all-cause mortality in patients with acute myocardial infarction—Data from Infarction Prognosis Study (IPS) Registry. Circ. J..

[54] Ninomiya T., Nagata M., Hata J., Hirakawa Y., Ozawa M., Yoshida D., Ohara T., Kishimoto H., Mukai N., Fukuhara M. (2013). Association between ratio of serum eicosapentaenoic acid to arachidonic acid and risk of cardiovascular disease: The Hisayama Study. Atherosclerosis.

[55] Nishizaki Y., Daida H. (2020). Optimal Dose of n-3 Polyunsaturated Fatty Acids for Cardiovascular Event Prevention. Circ. Rep..

[56] Marchioli R., Barzi F., Bomba E., Chieffo C., Di Gregorio D., Di Mascio R., Franzosi M.G., Geraci E., Levantesi G., Maggioni A.P. (1999). Dietary supplementation with n-3 polyunsaturated fatty acids and vitamin E after myocardial infarction: Results of the GISSI-Prevenzione trial. Gruppo Italiano per lo Studio della Sopravvivenza nell’Infarto miocardico. Lancet.

[57] Tavazzi L., Maggioni A.P., Marchioli R., Barlera S., Franzosi M.G., Latini R., Lucci D., Nicolosi G.L., Porcu M., Tognoni G. (2008). Effect of n-3 polyunsaturated fatty acids in patients with chronic heart failure (the GISSI-HF trial): A randomised, double-blind, placebo-controlled trial. Lancet.

[58] Yokoyama M., Origasa H., Matsuzaki M., Matsuzawa Y., Saito Y., Ishikawa Y., Oikawa S., Sasaki J., Hishida H., Itakura H. (2007). Effects of eicosapentaenoic acid on major coronary events in hypercholesterolaemic patients (JELIS): A randomised open-label, blinded endpoint analysis. Lancet.

[59] Kromhout D., Giltay E.J., Geleijnse J.M., Alpha Omega Trial Group (2010). N-3 fatty acids and cardiovascular events after myocardial infarction. N. Engl. J. Med..

[60] Galan P., Kesse-Guyot E., Czernichow S., Briancon S., Blacher J., Hercberg S., SU.FOL.OM3 Collaborative Group (2010). Effects of B vitamins and omega 3 fatty acids on cardiovascular diseases: A randomised placebo controlled trial. BMJ.

[61] Bosch J., Gerstein H.C., Dagenais G.R., Diaz R., Dyal L., Jung H., Maggiono A.P., Probstfield J., Ramachandran A., ORIGIN Trial Investigators (2012). N-3 fatty acids and cardiovascular outcomes in patients with dysglycemia. N. Engl. J. Med..

[62] Roncaglioni M.C., Tombesi M., Avanzini F., Barlera S., Caimi V., Longoni P., Marzona I., Milani V., Silletta M.G., Risk and Prevention Study Collaborative Group (2013). N-3 fatty acids in patients with multiple cardiovascular risk factors. N. Engl. J. Med..

[63] Bonds D.E., Harrington M., Worrall B.B., Bertoni A.G., Eaton C.B., Hsia J., Robinson J., Clemons T.E., Fine L.J., Writing Group for the AREDS2 Research Group (2014). Effect of long-chain omega-3 fatty acids and lutein + zeaxanthin supplements on cardiovascular outcomes: Results of the Age-Related Eye Disease Study 2 (AREDS2) randomized clinical trial. JAMA Intern. Med..

[64] Bowman L., Mafham M., Wallendszus K., Stevens W., Buck G., Barton J., Murphy K., Aung T., Haynes R., ASCEND Study Collaborative Group (2018). Effects of n-3 Fatty Acid Supplements in Diabetes Mellitus. N. Engl. J. Med..

[65] Manson J.E., Cook N.R., Lee I.M., Christen W., Bassuk S.S., Mora S., Gibson H., Albert C.M., Gordon D., Copeland T. (2019). Marine n-3 Fatty Acids and Prevention of Cardiovascular Disease and Cancer. N. Engl. J. Med..

[66] Nishizaki Y., Miyauchi K., Iwata H., Inoue T., Hirayama A., Kimura K., Ozaki Y., Murohara T., Ueshima K., Kuwabara Y. (2023). Study protocol and baseline characteristics of Randomized trial for Evaluation in Secondary Prevention Efficacy of Combination Therapy-Statin and Eicosapentaenoic Acid: RESPECT-EPA, the combination of a randomized control trial and an observational biomarker study. Am. Heart J..

[67] Mason R.P., Eckel R.H. (2021). Mechanistic Insights from Reduce-IT Strengthen the Case Against Triglyceride Lowering as a Strategy for Cardiovascular Disease Risk Reduction. Am. J. Med..

[68] Nosaka K., Miyoshi T., Iwamoto M., Kajiya M., Okawa K., Tsukuda S., Yokohama F., Sogo M., Nishibe T., Matsuo N. (2017). Early initiation of eicosapentaenoic acid and statin treatment is associated with better clinical outcomes than statin alone in patients with acute coronary syndromes: 1-year outcomes of a randomized controlled study. Int. J. Cardiol..

[69] Sherratt S.C.R., Libby P., Budoff M.J., Bhatt D.L., Mason R.P. (2023). Role of Omega-3 Fatty Acids in Cardiovascular Disease: The Debate Continues. Curr. Atheroscler. Rep..

[70] Ridker P.M., Rifai N., MacFadyen J., Glynn R.J., Jiao L., Steg P.G., Miller M., Brinton E.A., Jacobson T.A., Tardif J.C. (2022). Effects of Randomized Treatment with Icosapent Ethyl and a Mineral Oil Comparator on Interleukin-1beta, Interleukin-6, C-Reactive Protein, Oxidized Low-Density Lipoprotein Cholesterol, Homocysteine, Lipoprotein(a), and Lipoprotein-Associated Phospholipase A2: A REDUCE-IT Biomarker Substudy. Circulation.

[71] Olshansky B., Chung M.K., Budoff M.J., Philip S., Jiao L., Doyle R.T., Copland C., Giaquinto A., Juliano R.A., Bhatt D.L. (2020). Mineral oil: Safety and use as placebo in REDUCE-IT and other clinical studies. Eur. Heart J. Suppl..

[72] Sherratt S.C.R., Libby P., Bhatt D.L., Mason R.P. (2023). Comparative Effects of Mineral Oil, Corn Oil, Eicosapentaenoic Acid, and Docosahexaenoic Acid in an In Vitro Atherosclerosis Model. J. Am. Heart Assoc..

[73] Manubolu V.S., Budoff M.J., Lakshmanan S. (2022). Multimodality Imaging Trials Evaluating the Impact of Omega-3 Fatty Acids on Coronary Artery Plaque Characteristics and Burden. Heart Int..

[74] Budoff M.J., Bhatt D.L., Kinninger A., Lakshmanan S., Muhlestein J.B., Le V.T., May H.T., Shaikh K., Shekar C., Roy S.K. (2020). Effect of icosapent ethyl on progression of coronary atherosclerosis in patients with elevated triglycerides on statin therapy: Final results of the EVAPORATE trial. Eur. Heart J..

[75] Motoyama S., Nagahara Y., Sarai M., Kawai H., Miyajima K., Sato Y., Matsumoto R., Takahashi H., Naruse H., Ishii J. (2022). Effect of Omega-3 Fatty Acids on Coronary Plaque Morphology—A Serial Computed Tomography Angiography Study. Circ. J..

[76] Alfaddagh A., Elajami T.K., Ashfaque H., Saleh M., Bistrian B.R., Welty F.K. (2017). Effect of Eicosapentaenoic and Docosahexaenoic Acids Added to Statin Therapy on Coronary Artery Plaque in Patients With Coronary Artery Disease: A Randomized Clinical Trial. J. Am. Heart Assoc..

[77] Watanabe T., Ando K., Daidoji H., Otaki Y., Sugawara S., Matsui M., Ikeno E., Hirono O., Miyawaki H., Yashiro Y. (2017). A randomized controlled trial of eicosapentaenoic acid in patients with coronary heart disease on statins. J. Cardiol..

[78] Yamano T., Kubo T., Shiono Y., Shimamura K., Orii M., Tanimoto T., Matsuo Y., Ino Y., Kitabata H., Yamaguchi T. (2015). Impact of eicosapentaenoic acid treatment on the fibrous cap thickness in patients with coronary atherosclerotic plaque: An optical coherence tomography study. J. Atheroscler. Thromb..

[79] Nishio R., Shinke T., Otake H., Nakagawa M., Nagoshi R., Inoue T., Kozuki A., Hariki H., Osue T., Taniguchi Y. (2014). Stabilizing effect of combined eicosapentaenoic acid and statin therapy on coronary thin-cap fibroatheroma. Atherosclerosis.

[80] Niki T., Wakatsuki T., Yamaguchi K., Taketani Y., Oeduka H., Kusunose K., Ise T., Iwase T., Yamada H., Soeki T. (2016). Effects of the Addition of Eicosapentaenoic Acid to Strong Statin Therapy on Inflammatory Cytokines and Coronary Plaque Components Assessed by Integrated Backscatter Intravascular Ultrasound. Circ. J..

[81] Sugizaki Y., Otake H., Kuroda K., Kawamori H., Toba T., Nagasawa A., Takeshige R., Nakano S., Matsuoka Y., Tanimura K. (2020). Concomitant Use of Rosuvastatin and Eicosapentaenoic Acid Significantly Prevents Native Coronary Atherosclerotic Progression in Patients With In-Stent Neoatherosclerosis. Circ. J..

[82] Ahn J., Park S.K., Park T.S., Kim J.H., Yun E., Kim S.P., Lee H.W., Oh J.H., Choi J.H., Cha K.S. (2016). Effect of n-3 Polyunsaturated Fatty Acids on Regression of Coronary Atherosclerosis in Statin Treated Patients Undergoing Percutaneous Coronary Intervention. Korean Circ. J..

[83] Kita Y., Watanabe M., Kamon D., Ueda T., Soeda T., Okayama S., Ishigami K., Kawata H., Horii M., Inoue F. (2020). Effects of Fatty Acid Therapy in Addition to Strong Statin on Coronary Plaques in Acute Coronary Syndrome: An Optical Coherence Tomography Study. J. Am. Heart Assoc..

[84] Gencer B., Djousse L., Al-Ramady O.T., Cook N.R., Manson J.E., Albert C.M. (2021). Effect of Long-Term Marine ɷ-3 Fatty Acids Supplementation on the Risk of Atrial Fibrillation in Randomized Controlled Trials of Cardiovascular Outcomes: A Systematic Review and Meta-Analysis. Circulation.

[85] Kapoor K., Alfaddagh A., Al Rifai M., Bhatt D.L., Budoff M.J., Nasir K., Miller M., Welty F.K., McEvoy J.W., Dardari Z. (2021). Association between Omega-3 Fatty Acid Levels and Risk for Incident Major Bleeding Events and Atrial Fibrillation: MESA. J. Am. Heart Assoc..

